# Different role of circulating myeloid-derived suppressor cells in patients with multiple myeloma undergoing autologous stem cell transplantation

**DOI:** 10.1186/s40425-018-0491-y

**Published:** 2019-02-07

**Authors:** Sung-Eun Lee, Ji-Young Lim, Tae Woo Kim, Da-Bin Ryu, Sung Soo Park, Young-Woo Jeon, Jae-Ho Yoon, Byung-Sik Cho, Ki-Seong Eom, Yoo-Jin Kim, Hee-Je Kim, Seok Lee, Seok-Goo Cho, Dong-Wook Kim, Jong Wook Lee, Chang-Ki Min

**Affiliations:** 1 Department of Hematology, Seoul St. Mary’s Hospital, College of Medicine, The Catholic University of Korea, 222 Banpodae-ro, Seocho-gu, Seoul, 06591 Korea; 2 Leukemia Research Institute, The Catholic University of Korea, Seoul, Korea

**Keywords:** Myeloid-derived suppressor cells, Colony-stimulating factor 1 receptor, Multiple myeloma, Autologous stem cell transplantation

## Abstract

**Background:**

The aim of this study is to evaluate the prognostic impact of myeloid-derived suppressor cells (MDSCs) in multiple myeloma (MM) in the context of autologous stem cell transplantation (ASCT).

**Methods:**

Peripheral blood samples were collected for measuring monocytic (M-) MDSCs (CD14^pos^HLA-DR^low/neg^) and early-stage (E-) MDSCs (Lin^neg^HLA-DR^neg^CD33^pos^CD11b^pos^) before and after ASCT. Clinical outcomes following ASCT differed according to the frequency of each MDSC phenotype.

**Results:**

In the pre-ASCT analyses, lower M-MDSCs (<median) but not E-MDSCs were associated with a longer time to progression (TTP), whereas both MDSC phenotypes post-ASCT did not have a role in TTP. Both MDSC phenotypes pre-ASCT but not post-ASCT similarly suppressed in vitro autologous T and natural killer T cell proliferation. Importantly, pre-ASCT M-MDSCs more strongly inhibited the in vitro cytotoxic effect of melphalan compared with pre-ASCT E-MDSCs. Transcriptome analysis of each isolated MDSC subtype showed that expression of osteoclastic differentiation factors, particularly colony-stimulating factor 1 receptor (CSF1R), was significantly increased in M-MDSCs pre-ASCT. Finally, blockade of CSF1R substantially recovered the melphalan-induced cytotoxicity reduced by pre-ASCT M-MDSCs.

**Conclusions:**

Our data demonstrate that pre-ASCT M-MDSCs are correlated with poor clinical outcomes after ASCT through reduced cytotoxicity of melphalan. We propose that targeting CSF1R on these cells may improve the results of ASCT in MM.

**Electronic supplementary material:**

The online version of this article (10.1186/s40425-018-0491-y) contains supplementary material, which is available to authorized users.

## Introduction

For over two decades, autologous stem cell transplantation (ASCT) has been the standard consolidation treatment for transplant-eligible patients with newly diagnosed multiple myeloma (MM) to improve depth of response, progression-free survival (PFS), and likely overall survival (OS) [1]. Currently, MM represents the most common indication for ASCT as the front-line or delayed settings [2, 3]. Use of the immunomodulatory drugs thalidomide and lenalidomide and the proteasome inhibitor bortezomib before and after ASCT improves clinical outcomes [4, 5], although the relative impact of choice of pre-transplant induction and post-transplant therapy on long-term survival remains unknown [6]. Despite these clinical improvements, the vast majority of patients eventually experience disease relapse and progression.

Large numbers of myeloid-derived suppressor cells (MDSCs), a mixture of monocytic and granulocytic cells, accumulate during many pathologic conditions, including cancer, infectious diseases, trauma, and sepsis. MDSCs are characterized by myeloid origin, immature state, and most importantly by their potent ability to suppress different aspects of immune responses, especially T cell proliferation and cytokine production [7]. Currently, using specific markers, MDSCs can be phenotypically characterized. In humans, granulocytic MDSCs (G-MDSCs) are defined as lacking expression of CD14 but expressing CD15/CD33/CD11b, whereas monocytic-MDSCs (M-MDSCs) express CD14/CD11b and are characterized as HLA-DR^−/low^ cells or CD33^+^ cells [8]. Lin^−^ (including CD3, CD14, CD15, CD19, CD56) HLA-DR^−^CD33^+^ cells contain mixed groups of MDSC comprising more immature progenitors, which have been defined as early-stage MDSC (E-MDSCs) [9]. MDSCs not only inhibit anti-tumour immunity, but also directly stimulate tumorigenesis, tumour growth, and tumour expansion [10].

A growing body of evidence suggests that MDSCs offer an appealing target for therapeutic intervention in cancer treatment [11, 12]. Down-regulation of MDSC frequencies and/or abrogation of their immunosuppressive functions have been reported to delay tumour growth and prolong survival in both animal models and cancer patients [13, 14]. The emerging role of MDSCs in MM pathogenesis and clinical behaviour has been highlighted, and their increase in both peripheral blood (PB) and bone marrow (BM) of MM patients with bidirectional interaction between MDSCs and malignant plasma cells within the MM microenvironment has been documented [15–17]. The presence of inflammatory cytokines after high-dose chemotherapy leads to proliferation and activation of MDSCs originating from autologous hematopoietic progenitors at the time of engraftment. Therefore, each subset of MDSCs before and/or after transplant could be considered as a prognostic predictor as well as an important target contributing to MM progression in the context to ASCT. Here, we investigate clinical correlations and preclinical proof-of-concept data on the role of MDSCs in transplant outcomes and highlight the mechanistically relevant protection of MM against melphalan and the host immune system.

## Materials and methods

### Patients and transplant procedures

A total of 100 consecutive patients with MM who underwent ASCT as part of a front-line treatment at our institution between January 2013 and December 2016 were enrolled in this analysis. General ASCT procedures are summarized in the supplemental data (Additional file 1) [18].

### Blood sample collection and isolation of peripheral blood mononuclear cells (PBMCs)

Blood samples for the analysis of MDSC frequency were collected at diagnosis and pre- and post-ASCT. Pre-ASCT sampling was performed before conditioning chemotherapy, and post-ASCT sampling was done one day after neutrophil engraftment. PBMCs were freshly isolated from whole blood (30 mL) and were processed immediately for flow cytometric analysis.

### Flow cytometric analysis and isolation of MDSCs from PBMCs

MDSCs were phenotypically divided into two categories, M-MDSCs and E-MDSCs. E-MDSCs immunophenotyped as the HLA-DR^−^Lin^−^ CD11b^+^CD33^+^ population and M-MDSCs as the HLA-DR^−^CD14^+^ population were quantitated as a percentage of PBMCs (Additional file 4: Figure S1). Monoclonal antibodies for the identification of E- and M-MDSCs and isolation of MDSCs from PBMCs are summarized in the supplemental data (Additional file 1).

### Quantitative reverse transcription (qRT)-PCR analysis of MDSC RNAs

One microgram of total RNA was reverse transcribed into cDNA. Quantitative assessment of target mRNA levels was performed by real-time PCR with a CFX96 Real-Time PCR Detection System (Bio-Rad, Hercules, CA, USA). Primer sequences were as previously described (Additional file 2: Table S1) [19].

### T cell suppression assay

MDSCs and T cells were isolated from PBMCs of MM patients. Isolated MDSCs were cocultured with CFSE-labelled autologous T cells (MDSC:T cell ratio 1:1). T cell stimulation was provided by 2 μg/ml of anti-CD3/CD28 (eBioscience, San Diego, CA, USA) and 5 ng/ml of recombinant human IL-2 (R&D Systems, Minneapolis, MN, USA). After five days of incubation, the cells were stained with anti-CD4, anti-CD8, and anti-CD56 (eBioscience). Proliferation of T cells was analysed using LSRII (BD Pharmingen, San Jose, CA, USA) and Flowjo software (Ashland, OR, USA).

### Assay for apoptosis

CFSE-labelled IM-9, RPMI 8266, OPM2 cell lines and primary MM cells were cultured with or without isolated MDSCs (MM cell:MDSC ratio 1:1) in the presence of human M-CSF. The cocultured CFSE-positive cells were then incubated with or without 10 uM melphalan and 500 nM BLZ945 (Additional file 1). After incubation for 48 h, the cells were harvested, stained with Annexin V-APC and propidium iodide (PI), and examined by flow cytometry. Data obtained from flow cytometry were analysed using Flowjo software.

### Transcriptome sequencing and bioinformatics analysis

RNA extraction, cDNA library preparation, and bioinformatics analysis of the sequencing data are summarized in the supplemental data (Additional file 1).

### Definitions and statistical analysis

OS from transplantation was defined as the time from ASCT to death from any cause, and surviving patients were censored at the last follow-up. PFS was measured as the time from ASCT to disease progression or death (regardless of cause), whichever came first. We wanted to observe the effect of circulating MDSCs on disease progression after ASCT. Therefore, time to progression (TTP) was calculated as time from ASCT to disease progression, with deaths due to causes other than progression censored. Statistical analyses are summarized in the supplemental data (Additional file 1).

## Results

### Patients and transplant outcomes

A total of 100 patients, 59 males and 41 females, with a median age of 56 years (range, 33–67 years) were analysed in this study (Additional file 3: Table S2). Median disease duration before ASCT was 7.0 months (range, 2.9–12.3 months). The International Staging System (ISS) stages II, II, and III at diagnosis comprised 29, 44, and 23% of subjects, respectively, with 4% unknown ISS) [20]. After induction chemotherapy, 43 (43%), 35 (35%), and 22 (22%) patients had complete response, very good partial response (VGPR), and PR, respectively. The median follow-up was 36 months (95% CI, 30.6–42.5) for survivors. A total of 14 (14%) patients died, and 42 (42%) patients had disease progression. The 3-year OS and PFS were 84.8 ± 4.6% and 42.2 ± 6.3%, respectively (median OS and PFS were not reached and 26.6 months, respectively), and the 3-year TTP was 43.8 ± 6.3% (median TTP was 26.6 months).

### Changes in MDSCs during induction chemotherapy and ASCT

**Fig. 1:**
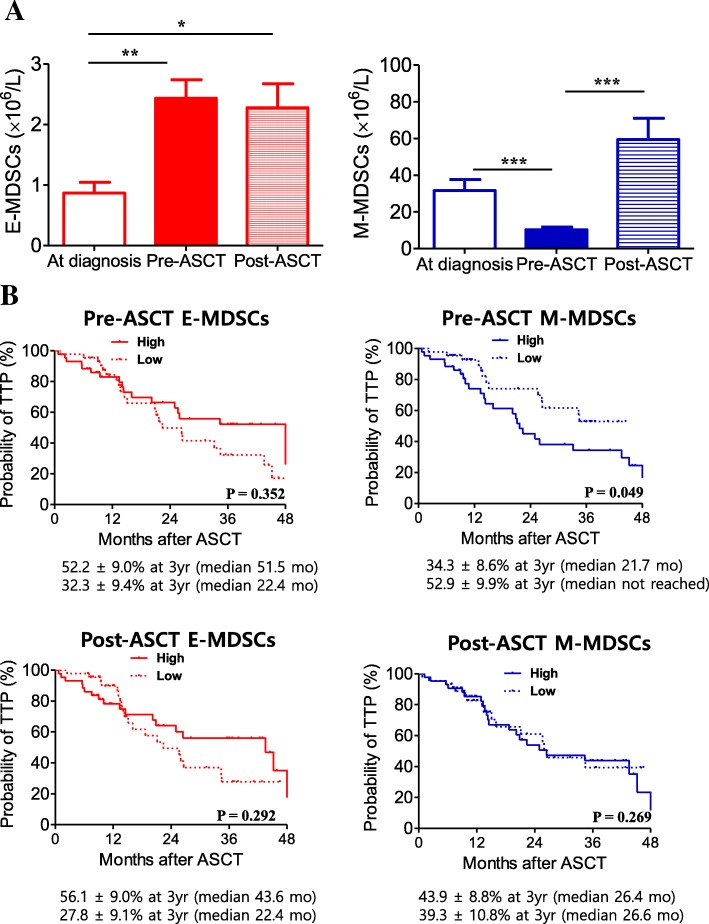
Clinical relevance of MDSCs during induction chemotherapy and ASCT. Serial changes in MDSC phenotypes through induction chemotherapy and ASCT (**a**). The data are presented as the mean ± SEM. *P < 0.05; **P < 0.01; ***P < 0.001. The 100 patients were grouped (low versus high) according to median frequency value of each E- (0.21 for pre-ASCT, 0.85 for post-ASCT) and M-MDSC phenotype (0.15 for pre-ASCT, 1.04 for post-ASCT). The 3-year time to progression (TTP) between the low and high pre-ASCT E-MDSC groups (**b**, top left) and M-MDSC groups (**b**, top right). The 3-year TTP according to post-ASCT MDSC phenotype groups are shown at the bottom

Figure 1a shows serial changes in MDSC phenotypes through induction chemotherapy and ASCT. At diagnosis, absolute number of E-MDSC phenotype was 0.9 ± 0.2 × 10^6^/L, which significantly increased to 2.4 ± 0.3 × 10^6^/L (*P* = 0.002) after induction chemotherapy. In contrast, absolute number of M-MDSC phenotype was significantly decreased after induction chemotherapy, from 31.6 ± 6.0 × 10^6^/L at diagnosis to 21.3 ± 4.6 × 10^6^/L (*P* < 0.001). When absolute numbers of pre-and post-ASCT MDSC phenotypes were compared, there was no difference in E-MDSC phenotypes (*P* = 0.757), whereas M-MDSC phenotype increased after ASCT (*P* < 0.001). The frequency of E-MDSC phenotypes at time of diagnosis was not significantly different among the three groups divided by the ISS, whereas a higher frequency of M-MDSCs was significantly associated with a higher ISS stage (Additional file 5: Figure S2).

### Expansion of MDSCs in relation to time to progression (TTP)

Next, we evaluated how each MDSC phenotype in both pre- and post-ASCT correlated with the 3-year TTP. The patients were grouped according to median frequency value of each MDSC phenotype. First, the association of pre-ASCT MDSCs with the 3-year TTP was analysed and showed that there was no difference between the high and low E-MDSCs groups (52.2% vs. 32.3%, *P* = 0.352) (Fig. 1b, top left). In contrast to the E-MDSCs groups, the 3-year TTP was significantly lower in the high M-MDSC group compared with the low M-MDSC group (34.3 vs. 52.9, *P* = 0.049, Fig. 1b top right). Second, we also analysed the effect of post-ASCT MDSCs on the 3-year TTP, which showed that neither E- or M-MDSC phenotype correlated with the 3-year TTP (Fig. 1b, bottom). Ultimately, after adjusting for potential risk factors (immunoglobulin type and serum calcium level at diagnosis), multivariate analysis revealed that the high M-MDSC group pre-ASCT was associated with a lower TTP, with an HR of 0.49 (95% CI, 0.24 to 0.99, *P* = 0.045) (Table 1).

**Table 1:** Predictive factors for time to progression

Univariate analysis	RR (95% CI)	*P*
Age at diagnosis (years), continuous	1.00 (0.96–1.04)	0.855
Sex (F vs. M)	0.92 (0.50–1.71)	0.802
Durie-Salmon stage at diagnosis (III vs. II)	1.29 (0.54–3.08)	0.567
ISS stage at diagnosis (III vs. I-II)	0.64 (0.29–1.39)	0.257
Cytogenetics (high risk vs. standard)	1.81 (0.80–4.08)	0.155
Immunoglobulin type (others vs. light chain only)	2.27 (1.04–4.93)	0.039
Myeloma bone disease on plain radiographs (no vs. yes)	1.69 (0.89–3.919)	0.107
Cr at diagnosis (mg/dL), (≥2 vs. < 2)	0.61 (0.29–1.28)	0.189
Hb at diagnosis (g/dL), (≥8.5 vs. < 8.5)	0.58 (0.31–1.08)	0.084
Ca at diagnosis (mg/dL), (≥10 vs. < 10)	1.01 (0.49–2.07)	0.985
β2-microglobulin at diagnosis (mg/dL), (≥5.5 vs. < 5.5)	0.60 (0.28–1.31)	0.199
Albumin at diagnosis (mg/dL), (≥3.5 vs. < 3.5)	0.58 (0.31–1.08)	0.083
LDH at diagnosis (U/L), (≥450 vs. < 450)	1.20 (0.60–2.43)	0.607
Multivariate analysis	RR (95% CI)	P
Immunoglobulin type (others vs. light chain only)	2.01 (0.77–5.24)	0.153
Hb at diagnosis (g/dL), (≥8.5 vs. < 8.5)	0.79 (0.40–1.58)	0.507
Albumin at diagnosis (mg/dL), (≥3.5 vs. < 3.5)	0.60 (0.30–1.020)	0.148
Pre-ASCT M-MDSC frequency (Low vs. high)	0.49 (0.24–0.99)	0.045

*Ca* Calcium, *Cr* Creatinine, *CI* Confidence interval, *F* Female, *Hb* Hemoglobin, *LCD* Light chain disease, *LDH* Lactate dehydrogenase, *M* Male, *TTP* Time to progression

### Functional characterization of pre- and post-ASCT MDSCs

**Fig. 2:**
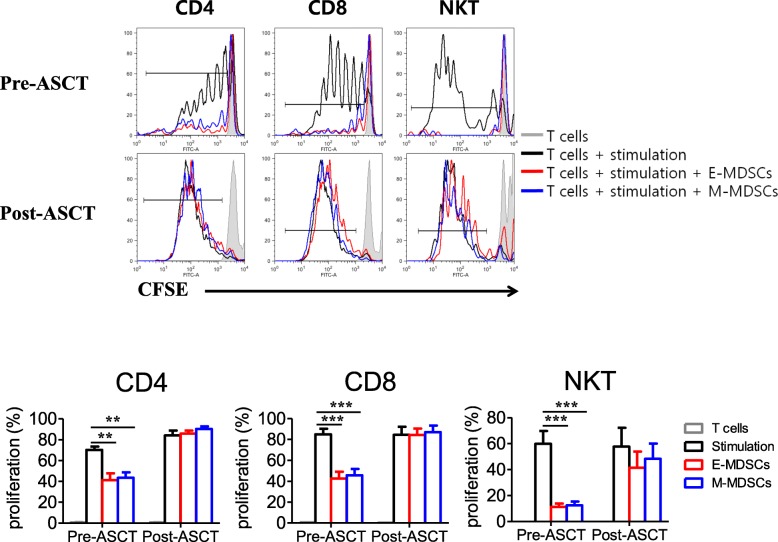
Suppressive function of pre- and post-ASCT MDSC phenotypes. We isolated E- and M-MDSC phenotypes from six patients’ PBMCs collected pre- and post-ASCT and tested autologous CD4, CD8 T-, and NKT-cell suppression mediated by each MDSC phenotype. The top figures are representative and individual data from independent experiments using MDSC phenotypes isolated from the six patients, as shown in the bottom figure. Both pre-ASCT E- and M-MDSC subsets had similarly suppressed autologous CD4 (left), CD8 T- (middle), and NKT-cell (right) proliferation. In contrast, post-ASCT E- and M-MDSC phenotypes did not show suppressive effects on those immune cells. The data are presented as the mean ± SEM. **P < 0.01; ***P < 0.001

To investigate the functional characterization of each MDSC phenotype in both pre- and post-ASCT, we isolated E- and M-MDSC phenotypes from patients’ PBMCs collected pre- and post-ASCT. And then, we tested autologous T- and NKT-cell suppression mediated by each MDSC phenotype (Fig. 2). Both pre-ASCT E- and M-MDSC phenotypes had similarly suppressed autologous T- and NKT- cell proliferation. In contrast, E- and M-MDSC phenotypes post-ASCT did not show suppressive effects on autologous T- and NKT- cells, which indicates these cells are not MDSCs but rather monocytes. It has been shown that MM-associated macrophages protect MM cells from chemotherapy drug-induced apoptosis in vitro [21]. M2-polarized macrophages also mainly upregulate CD200R and CD206 and downregulate CD14 [22]. CD200R and CD206 were expressed in M2 macrophages but not in pre-transplant isolated MDSC phenotypes (Additional file 6: Figure S3).

### Role of each MDSC phenotype in melphalan-induced cytotoxic activity

**Fig. 3:**
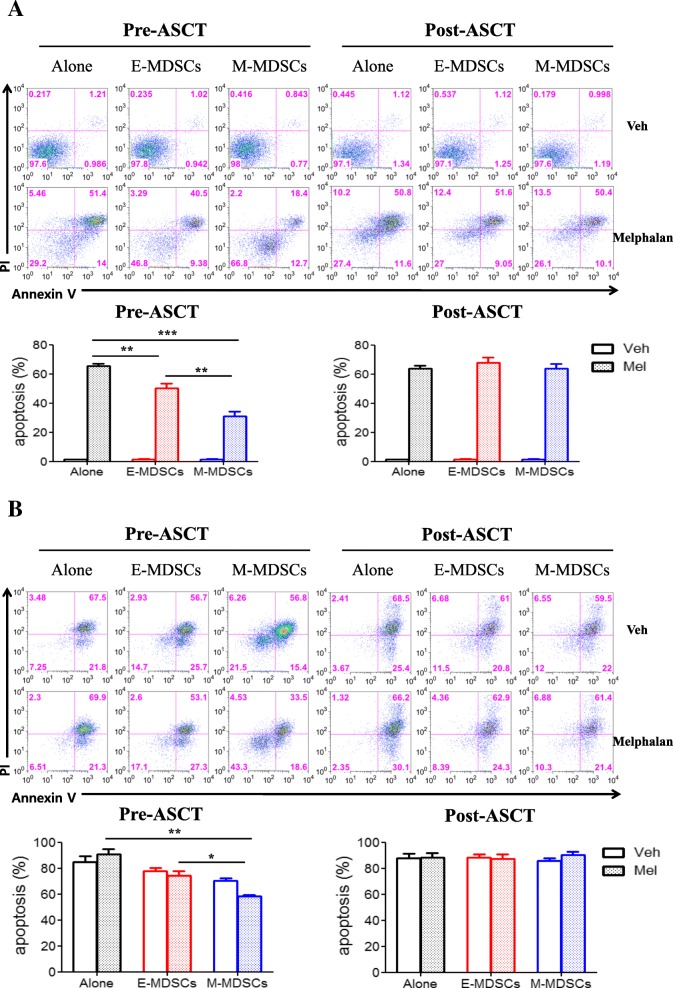
The influence of pre- and post-ASCT MDSC phenotypes on in vitro melphalan-induced cytotoxic assay. MM cell line, IM-9 cells (**a**) or primary MM cells (**b**) were cultured with or without MDSCs isolated from pre- and post-ASCT samples (MM cell:MDSC ratio 1:1) in the presence of human M-CSF. The top figures are representative staining with Annexin V-APC and PI after incubation with or without melphalan. In the bottom figure, individual data from independent melphalan-induced cytotoxic assay by E- and M-MDSC phenotypes isolated from five patients were compared. The label of post-ASCT MDSCs on the figure means the cells expressing each MDSC phenotype. The data are presented as the mean ± SEM. *P < 0.05; **P < 0.01; ***P < 0.001

To better understand the mechanisms leading to poor prognosis mediated by the pre-ASCT, but not post-ASCT, M-MDSC phenotype, we tested the influence of E- and M-MDSCs isolated from patient PBMCs on in vitro melphalan-induced cytotoxic assay according to time before and after ASCT. First, in the test using the MM cell line (IM-9) (Fig. 3a), pre-ASCT M-MDSCs inhibited melphalan-induced cytotoxic effects more strongly than pre-ASCT E-MDSCs. In contrast, isolated cells of M- and E-MDSC phenotype post-ASCT did not have any inhibitory effect on melphalan-induced cytotoxic activity. Next, primary CD138^+^ cells taken from patients’ BM were examined for melphalan-induced cytotoxicity in the presence of E- and M-MDSCs isolated from another patient at the time before and after ASCT (Fig. 3b). Similarly, pre-ASCT M-MDSCs were capable of reducing the cytotoxic activity of melphalan on primary myeloma cells more strongly than pre-ASCT E-MDSCs, whereas the post-ASCT MDSC phenotypes did not show the inhibitory effect.

### Differentially expressed genes between E-MDSC and M-MDSC phenotypes before and after ASCT

**Fig. 4:**
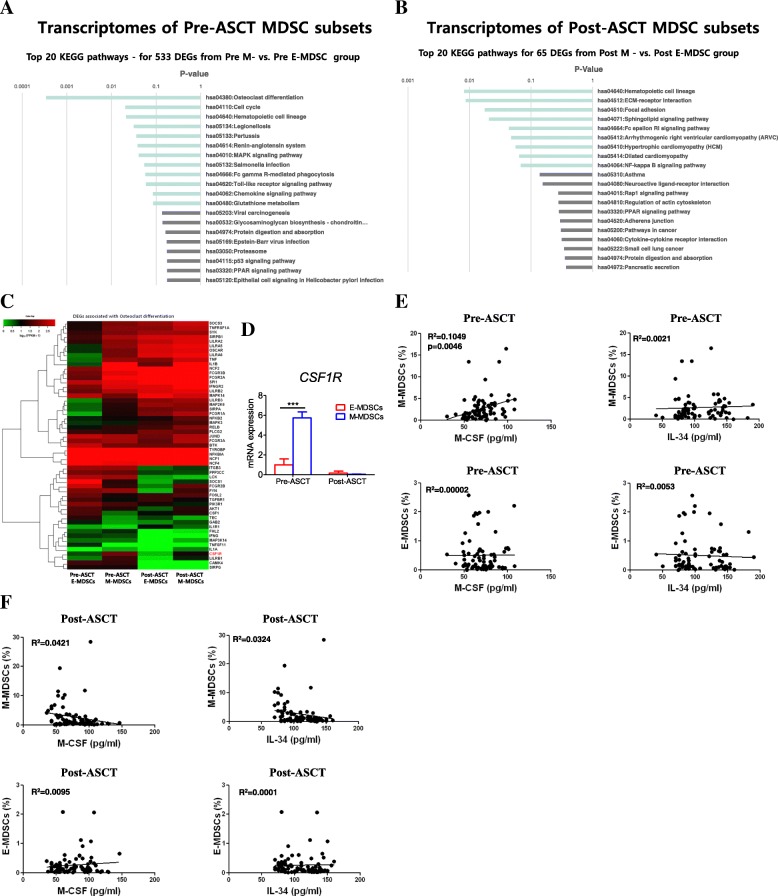
Transcriptome profiling analysis of isolated E- and M-MDSC phenotypes. The top 20 KEGG pathways for 533 differentially expressed genes between pre-ASCT E- and M-MDSC populations (**a**) and for 65 differentially expressed genes between post-ASCT E- and M-MDSC phenotypic populations (**b**), using a threshold of a 2-fold change and *P*-value < 0.05. The most remarkable difference was osteoclast differentiation in pre-ASCT M- versus E-MDSCs, which was not observed in post-ASCT M- versus E-MDSC phenotypes. Among the genes associated with osteoclast differentiation, *CSF1R* was the most significant (**c**) and was confirmed using qRT-PCR in isolated peri-ASCT E- and M-MDSC phenotypes (**d**). The data are presented as the mean ± SEM. *P < 0.05. Next, M-CSF and IL-34, which are known to trigger *CSF-1R* signalling in patient sera (*n* = 75 for M-CSF, *n* = 82 for IL-34), were measured, and the correlation between these factors and the frequency of pre-ASCT (**e**) and post-ASCT (**f**) MDSC phenotypes was analysed. The Spearman correlation coefficient was used to evaluate association for continuous variables. The label of post-ASCT MDSCs on the figure means the cells expressing each MDSC phenotype

As we found a negative impact of pre-ASCT M-MDSCs on TTP and in vitro melphalan-induced cytotoxicity, we were interested in which genes were differentially expressed between pre-ASCT E- and M-MDSCs. Using transcriptome resequencing, we analysed KEGG pathways for 533 differentially expressed genes between E- and M-MDSC populations using a threshold of a 2-fold change and *P*-value < 0.05. We found that the most remarkable difference was osteoclast differentiation in pre-ASCT M-MDSCs versus E-MDSCs (Fig. 4a). In contrast, no difference in expression of osteoclast differentiation was observed between post-ASCT E- and M-MDSC phenotypes (Fig. 4b). Next, we investigated the differentially expressed genes associated with osteoclast differentiation. Among them, *CSF1R* was a highly expressed gene in pre-ASCT M-MDSCs compared to other phenotypes of MDSCs (Fig. 4c). These results were confirmed using qRT-PCR in isolated peri-ASCT E- and M-MDSC phenotypes. mRNA expression of *CSF1R* was much higher in pre-ASCT M-MDSCs (*n* = 9) than pre-ASCT E-MDSCs (*n* = 8) (*P* < 0.001; Fig. 4d, left), whereas there was no difference between post-ASCT M- (*n* = 11) and E-MDSC phenotypes (*n* = 12) (Fig. 4d, right).

### Relationships between serum levels of CSF1R ligands and circulating MDSC frequencies

Next, we measured factors such as M-CSF and IL-34, which are known to trigger CSF-1R signalling in patient sera (*n* = 75 for M-CSF, *n* = 82 for IL-34) [23]. In correlation analysis between these factors and the frequency of pre-ASCT MDSCs, M-CSF was correlated with M-MDSCs frequency (R^2^ = 0.1049, *P* = 0.005), verifying that M-CSF enhanced M-MDSCs proliferation (Fig. 4e, top left), but IL-34 did not correlate with M-MDSCs (Fig. 4e, top right). On the other hand, there was no relationship between these factors and the frequency of pre-ASCT E-MDSCs (Fig. 4e, bottom). Furthermore, neither post-ASCT E- (Fig. 4f, top) nor M-MDSC phenotype (Fig. 4f, bottom) was related to level of M-CSF or IL-34.

### CSF1R signalling is critical for attenuation of melphalan-induced cytotoxic effect by pre-ASCT M-MDSCs

**Fig. 5:**
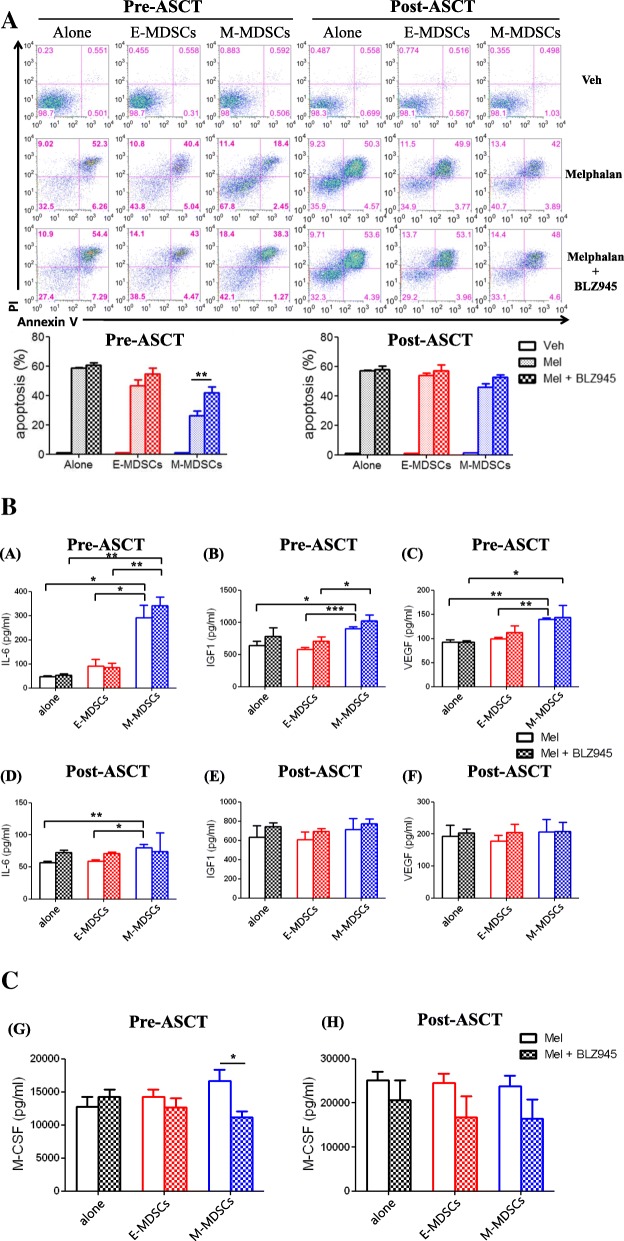
The influence of CSF1R inhibition on melphalan-induced cytotoxicity attenuated by pre-ASCT M-MDSCs. The influence of BLZ945, a human CSF1R inhibitor, on cell apoptosis induced by melphalan was tested (**a**). IM-9 cells were cultured with or without MDSCs isolated from pre- and post-ASCT patients (MM:MDSC ratio 1:1) in the presence of human M-CSF, as shown in Fig. 3. The top figures are representative staining with Annexin V-APC and PI after incubation with vehicle, 10 uM melphalan with or without 500 nM BLZ945. In the bottom figure, individual data from independent experiments by E- and M-MDSC phenotypes isolated from five patients were compared. (**b**) Cytokines (IL-6, IGF1, VEGF, and M-CSF) in culture supernatants with pre- and post-ASCT MDSC phenotypes were measured, and the effects of BLZ945 treatment were compared. The concentrations of IL-6, IGF1, and VEGF in culture supernatants with pre-ASCT MDSCs (top) and post-ASCT MDSC phenotypes (bottom) are shown. (**c**) M-CSF concentrations in culture supernatants with pre-ASCT MDSCs (left) and post-ASCT MDSC phenotypes (right) are shown. MDSC phenotypes were isolated from six patients. The label of post-ASCT MDSCs on the figure means the cells expressing each MDSC phenotype. The data are presented as the mean ± SEM. *P < 0.05; **P < 0.01; ***P < 0.001

Finally, to determine whether a CSF1R inhibitor can recover melphalan-induced cytotoxicity attenuated by pre-ASCT M-MDSCs, we examined the influence of BLZ945, a human CSF1R inhibitor, on cell death induced by melphalan (Fig. 5a). Presence of CSF1R inhibitor reversed the protective effect of pre-ASCT M-MDSCs on IM-9 cells. However, BLZ945-treated pre-ASCT E-MDSCs did not affect survival of the MM cells. The effect of post-ASCT E- and M-MDSC phenotypes on melphalan-induced cytotoxicity was not affected by BLZ945 treatment. Similar results were obtained using the RPMI 8226 and OPM2 cell lines (Additional file 7: Figure S4). Taken together, these results demonstrate that inhibition of CSF1R signalling results in recovery of anti-MM activity by melphalan, which is attenuated by pre-ASCT M-MDSCs. In addition, we measured several cytokines in the culture supernatants and compared them according to BLZ945 treatment because cytokines are major proliferative factors for malignant plasma cells. Although the concentrations of IL-6, IGF1, and VEGF with pre-ASCT M-MDSCs were higher than those with pre-ASCT E-MDSCs, BLZ945 treatment did not have an effect on their concentrations in the presence of pre-ASCT M-MDSC phenotypes (Fig. 5b, top). Concentrations of those cytokines in culture supernatants were not changed in the presence of post-ASCT MDSC phenotypes (Fig. 5b, bottom). Importantly, only M-CSF concentration was significantly decreased in the culture supernatants with pre-ASCT M-MDSCs after BLZ945 treatment (Fig. 5c).

## Discussion

The objective of this study is not only to provide a comprehensive analysis of MDSC biology in patients with MM undergoing ASCT, but also to suggest molecular and functional mechanisms for the effects of MDSCs on transplant outcomes. MDSCs have emerged as major regulators in diseases that involve chronic inflammation, especially cancer, but also infection, autoimmune diseases, trauma, graft-versus-host disease, and others. Although evidence of the clinical significance of MDSCs in cancer has emerged, previous studies have several limitations in that phenotypic characterization for human MDSCs is heterogeneous, and functional analyses of immunoregulatory activity are often lacking for practical reasons, mainly related to the paucity of MDSCs in human samples [9]. With recent studies, the cellular nature of human MDSCs has been better defined as G-MDSCs, M-MDSCs, and E-MDSCs [9, 24]. However, due to lack of unique phenotypic markers, suppressive activity is still important to characterize MDSCs from other cells referred to as tumour-associated neutrophils or monocytes [25, 26]. In this study, we showed for the first time that two main subgroups of MDSCs, E- and M-MDSCs, differentially affected clinical outcomes following ASCT. In the pre-ASCT analyses, higher M-MDSCs but not E-MDSCs were associated with a lower TTP, whereas neither MDSC phenotype post-ASCT had a role in TTP. Both MDSC subtypes pre-ASCT but not post-ASCT similarly suppressed in vitro autologous T and natural killer T cell proliferation. Therefore, according to established definition [9], phenotypic post-ASCT M-MDSCs and E-MDSCs are not MDSCs but rather monocytes and progenitors, respectively. Importantly, pre-ASCT M-MDSCs more potently inhibited in vitro cytotoxic effects of melphalan compared with pre-ASCT E-MDSCs. Until now, although the important attribute of immune-suppressive activity of MDSCs is known well, the potential targets on these cells responsible for poor clinical outcomes after ASCT remain to be fully characterized. By more sophisticated biochemical and transcriptome analysis of each isolated MDSC subtype, we showed that expression of osteoclastic differentiation factors, in particular *CSF1R*, was significantly increased in M-MDSCs pre-ASCT. Finally, our finding, in which blockade of *CSF1R* recovers the melphalan-induced cytotoxicity weakened by pre-ASCT M-MDSCs, allow us to take the next step in therapeutically targeting MDSCs in patients with MM undergoing ASCT.

The correlation between the systemic expansion of MDSCs and clinical outcome has been reported for both solid and hematologic human malignancies, confirming that MDSCs can influence tumour growth and metastases [10, 27]. Previous studies have investigated the clinical relevance of MDSCs in terms of tumour burden and clinical stage [28], sensitivity to chemo- and immunotherapy [29], and association with patient survival [30] in various types of cancer. Tumor-promoting and immune-suppressive roles of MDSCs in the MM microenvironment are also emerging [31]. Görgün et al. reported that MDSCs are increased in patients with MM and have bidirectional interaction with tumours within the MM microenvironment [15]. MDSCs from MM patients promote MM tumour growth and induce immune suppression; conversely, MM cells induce MDSC development. Other studies have also reported the presence and activation of MDSCs in MM patients [32, 33]. Because the direct actions and functional consequences of MDSCs on MM cells, especially in the context of ASCT, are poorly defined, our results further extend the understanding of the role of MDSCs and development of therapeutic strategies to target MDSCs in patients with MM undergoing ASCT.

In terms of the suppressive mechanisms of MDSCs in cancer patients, MDSCs have been found to employ a range of different cellular and molecular suppressive strategies. These mechanisms included Treg induction [34], ROS [35, 36], arginase [15, 37], TGF-β [38, 39], and the overlapping PGE2/COX-2/STAT3 pathways [37, 40, 41]. As suggested in our study, influence of circulating M-MDSCs on clinical outcomes has been commonly reported in cancer patients. On the other hand, some studies have reported significantly higher level of G-MDSCs in cancer patients compared with healthy individuals, and Ramachandran et al. demonstrated that G-MDSCs protected MM cells from chemotherapy [42]. The differential effect of MDSC subtypes on cancer cells should be interpreted cautiously because of the possibility of ambiguity in definition of MDSC subtypes across previously reported studies. In our current study, both MDSC phenotypes pre-ASCT but not post-ASCT had similarly suppressed in vitro autologous T and natural killer T cell proliferation. These results suggest that pre-ASCT M-MDSCs have a similar nature to those previously reported in cancer patients, whereas their characteristics were different from post-ASCT MDSC phenotypes, which were expanded from autologous peripheral blood progenitors as a secondary inflammatory response. In our previous study, both MDSC subtypes isolated early after allogeneic SCT had a capacity to suppress T cell proliferation, suggesting that alloimmune response greatly contributes to the immunosuppressive effect of MDSCs [19].

Despite the advent of novel agents and doubling of survival rates, MM is still considered an incurable malignancy [43], and ASCT is still the first-line treatment for transplant eligible patients [44]. MM is characterized by generalized immune suppression that contributes to susceptibility to infection, as well as tumour progression [45] and bidirectional interaction between malignant plasma cells and the BM microenvironment, which has a substantial role in chemotherapy resistance and thereby the persistence of residual disease [46, 47]. Therefore, to improve the efficiency of ASCT, we highlight MDSCs as an important target for therapeutics for patients with MM. Interestingly, we found that blockade of *CSF1R* recovered melphalan-induced cytotoxicity reduced by pre-ASCT M-MDSCs, which suggests that targeting *CSF1R* on M-MDSCs pre-ASCT may improve the results of ASCT in MM. Strategies for overcoming MDSC-mediated immune suppression have so far focused on reducing their level, inhibiting their suppressive function, or influencing their differentiation. Ramachandran et al. showed that growth of immunogenic MM cells was significantly reduced in S100A9KO mice, which are deficient in their ability to accumulate MDSCs in tumour-bearing hosts [17]. However, whether MDSC-mediated suppression in MM can be abrogated using inhibition of the possible suppressive pathway remains to be studied. Recently, Wang et al. explored the potential of targeting myeloma-associated macrophages using CSF1R-blocking mAb in mice suggesting that this approach may sensitize myeloma cells to chemotherapy and promote anti-myeloma immune responses. [48]. On the other hand, we focused on the effect of targeting human pre-ASCT M-MDSCs using the CSF1R blockade BLZ945. Our data are consistent with other studies showing that CSF1R blockade by inhibitors and antibodies improves therapeutic efficacy in various solid cancers [49, 50]. These findings support the possibility of repositioning of CSF1R blockade by inhibitors and antibodies into MM therapy in the context of ASCT. However, in this study, in vivo preclinical tests were not performed and characteristics of malignant plasma cells, such as cytogenetic abnormalities, were not considered. It is therefore unknown whether MDSC-targeted therapies will bring clinical benefit to patients. Further studies are needed to confirm the efficacy of CSF1R blockade by inhibitors and antibodies in patients with MM undergoing ASCT.

## Conclusions

In summary, we demonstrated that pre-ASCT M-MDSCs correlate with poor clinical outcomes after ASCT through reduced melphalan efficacy and propose that targeting *CSF1R* on these cells may improve the ASCT outcomes in MM. Although it is not known whether targeting *CSF1R* shows selective suppression against M-MDSCs in vivo, this study shows a possible strategy for overcoming M-MDSC-mediated reduction of melphalan effect in MM patients undergoing ASCT. Future studies should attempt to prove efficacy and safety in clinics.

## Additional files

Additional file 1: Supplementary Materials and Methods. (DOCX 24 kb)

Additional file 2: **Table S1.** Primers used for qPCR amplification. (DOCX 19 kb)

Additional file 3: **Table S2.** Baseline characteristics of patients. (DOCX 22 kb)

Additional file 4: **Figure S1.** Representative immunophenotypes of E- and M-MDSCs from PBMCs. Representative FACS plots of E- and M-MDSC phenotypes in PBMCs taken at the time of engraftment after ASCT. (TIF 180 kb)

Additional file 5: **Figure S2.** Correlation between the frequency of E- and M-MDSC phenotypes at diagnosis and disease stage by the International Staging System. The frequency of E- and M-MDSC phenotypes at diagnosis was compared in the three groups (*n* = 56, 93, and 79 for stage I, II, and III, respectively). The data are presented as the mean ± SEM. *P < 0.05. (TIF 36 kb)

Additional file 6: **Figure S3.** Different macrophage gene expression between M2 polarized macrophages and pre-ASCT MDSCs. M2 macrophages, E- and M-MDSCs were isolated from PBMCs in pre-ASCT (*n* = 9). Expression of CD200R and CD206 was assessed in M2 macrophages and pre-transplant isolated MDSCs by qRT-PCR. (TIF 34 kb)

Additional file 7: **Figure S4.** The influence of CSF1R inhibition on melphalan-induced cytotoxicity attenuated by pre-ASCT M-MDSCs was assessed in the RPMI 8266 (a) and OPM2 cell lines (b). The same procedure as in Fig. 5 was carried out. (PDF 1259 kb)

## Abbreviations

ASCT: Autologous stem cell transplantation
BM: Bone marrow
E-MDSCs: Early-stage MDSCs
G-MDSCs: Granulocytic MDSCs
ISS: International staging system
MDSCs: Myeloid-derived suppressor cells
MM: Multiple myeloma
M-MDSCs: Monocytic MDSCs
OS: Overall survival
PB: Peripheral blood
PBMCs: Peripheral blood mononuclear cells
PFS: Progression-free survival
qRT-PCR: Quantitative reverse transcription-PCR
TTP: Time to progression
VGPR: Very good partial response
