# Supplementary material for: Different role of circulating myeloid-derived suppressor cells in patients with multiple myeloma undergoing autologous stem cell transplantation
Source: J Immunother Cancer. 2019 Feb 7;7:35. doi: 10.1186/s40425-018-0491-y (PMC6367772; doi:10.1186/s40425-018-0491-y)
Supplement: Supplementary file 1 — Supplementary Materials and Methods. (DOCX 24 kb) [file 40425_2018_491_MOESM1_ESM.docx]

**Supplementary Materials and Methods**

**Transplant procedures**

ASCT was performed after achieving a response greater than a partial response (PR). All patients were mobilized with cyclophosphamide (3 g/m^2^ total) for two days and then treated subcutaneously once daily with G-CSF (lenograstim; JW Pharmaceutical, Seoul, Korea) at 10 μg/kg/day. Conditioning consisted of melphalan (100 mg/m^2^) for two days except in patients with serum creatinine above 2.0 mg/dl or on haemodialysis, who received 70 mg/m^2^ for two days. G-CSF (5 μg/kg/day) was administered subcutaneously to all patients from one day after transplantation until the absolute neutrophil count (ANC) reached >3.0 x 10^9^/L. All patients received prophylactic ciprofloxacin and an antifungal agent (micafungin) starting four days before transplantation and continued to receive these drugs until ANC reached 1.0 x 10^9^/L. Written informed consent was obtained from each patient before participation in this study. This study was approved by the Institutional Review Board of The Catholic University of Korea and was conducted in accordance with the Declaration of Helsinki.

**Isolation of MDSCs from PBMCs**

To purify M-MDSCs, PBMCs were purified using HLA-DR negative separation (Miltenyi Biotec, Auburn, CA, USA) and CD14 positive separation microbeads (Miltenyi Biotec). To purify E-MDSCs, the HLA-DR^-^CD14^-^ cell fraction was incubated with CD33 microbeads (Miltenyi Biotec) for positive selection of HLA-DR^-^CD33^+^ cells.

**RNA extraction and quantitative reverse transcription (qRT)-PCR analysis**

The freshly isolated MDSC samples were suspended in TRIzol reagent (Invitrogen, CA, USA), and the total RNA was immediately extracted according to the manufacturer’s instructions. The freshly extracted total RNA was stored at -80°C for subsequent testing. One microgram of total RNA was reverse transcribed into cDNA. Quantitative assessment of target mRNA levels was performed by real-time PCR with a CFX96 Real-Time PCR Detection System (Bio-Rad, Hercules, CA, USA). Primer sequences are given in Additional file 2: Table S1. The quantity of mRNA was calculated using the 2^-ΔΔCt^ method, and GAPDH was used to normalize total RNA quantities.

**Cell cultures and reagents**

Human MM cell lines (IM-9, RPMI 8266 and OPM2) were obtained from ATCC (Manassas, VA, USA). The MM cell lines and primary MM cells were cultured in RPMI-1640 medium supplemented with 10% FBS and 1% penicillin-streptomycin-glutamine solution (Invitrogen, Carlsbad, CA, USA). Melphalan was purchased from Sigma (St. Louis, MO, USA), human recombinant M-CSF from R&D Systems, and the CSFR1 inhibitor BLZ945 from SelleckChem (Houston, TX, USA).

For apoptosis assay, CFSE- labeled MM cells were co-cultured with or without isolated MDSCs. Only cultured MM cells were positive for CFSE, whereas MDSCs cultured alone showed CFSE negative. So, CFSE positive cells were used to examine the apoptosis of MM cells.

**RNA extraction, cDNA library preparation, and bioinformatics analysis of the sequencing data**

The sequencing library was prepared using the Illumina® TruSeq™ RNA Sample Preparation Guide, and was sequenced using an Illumina HiSeq 2000 platform following the manufacturer’s protocols. The sequencing reads were mapped against hg19 using STAR (v2.5), an ultrafast universal RNA-seq aligner35. All parameters were set to the default values except for the allowed maximum mismatch, which was set to 5% for each read, and the output of the BAM files, which was sorted by coordinate. The transcripts were assembled de novo using Cufflinks (v2.2.1)13, and the novel GTF file was merged with the GTF file of hg19. With the merged GTF file, the FPKM of each transcript was estimated using the Cuffquant and Cuffnorm tools contained in the Cufflinks suite. The differential expression of each transcript and each gene was analysed using Cuffdiff, also a component of Cufflinks. Condition-specific genes were also identified with CummeRbund by estimating the Jensen-Shannon distance. Gene Ontology (GO) and Kyoto Encyclopedia of Genes and Genomes (KEGG) pathway enrichment analyses were performed on the identified DEG using Database for Annotation, Visualization, and Integrated Discovery (DAVID, v6.7).

**Measurements of cytokines by ELISA**

Concentrations of IL-34 and M-CSF were measured in patient sera, and those IL-6, IGF1, VEGF, and M-CSF were analysed in supernatants of cocultures by ELISA. Assays were performed according to the manufacturer’s protocol (R&D Systems). ELISA plates were read using a microplate reader (Bio-Rad).

**Statistical analysis**

Statistical comparisons between groups were performed using the 2-trailed Student’s *t*-test for continuous variables. Survival curves were plotted according to the Kaplan and Meier method, and the log-rank test was used to assess potential prognostic factors. Cox proportional hazard regression model was used for multivariate analysis for TTP. Prognostic factors with a *P* value less than 0.1 in univariate analysis for TTP were entered into multivariate analysis. All *P* values were two-sided, and 5% was chosen as the level of statistical significance.
