# Supplementary material for: Different role of circulating myeloid-derived suppressor cells in patients with multiple myeloma undergoing autologous stem cell transplantation
Source: J Immunother Cancer. 2019 Feb 7;7:35. doi: 10.1186/s40425-018-0491-y (PMC6367772; doi:10.1186/s40425-018-0491-y)
Supplement: Supplementary file 2 — Table S1. Primers used for qPCR amplification. (DOCX 19 kb) [file 40425_2018_491_MOESM2_ESM.docx]

**Table S2. Primers used for qPCR amplification.**

| Gene | Forward sequence (5′-3′) | Reverse sequence (5′-3′) |  |
| --- | --- | --- | --- |
| CD200R  CD206 | TGGGAGGTCCACAATGTGTCTA  GCGATTAATAACAGCTAGTGGAAG | TGTACAGACTCTTGTTGCCAGTCA  TTCTCCATAAGCCCAGTTTTCA |  |
| GAPDH | ACC CAC TCC TCC ACC TTT GA | CAT ACC AGG AAA TGA GCT TGA CAA |  |
