# Supplementary material for: Different role of circulating myeloid-derived suppressor cells in patients with multiple myeloma undergoing autologous stem cell transplantation
Source: J Immunother Cancer. 2019 Feb 7;7:35. doi: 10.1186/s40425-018-0491-y (PMC6367772; doi:10.1186/s40425-018-0491-y)
Supplement: Supplementary file 3 — Table S2. Baseline characteristics of patients. (DOCX 22 kb) [file 40425_2018_491_MOESM3_ESM.docx]

**Table S1. Baseline characteristics of patients**

| **Characteristics** | **All patients**  **(N = 100)** |
| --- | --- |
| Age, years, median (range) | 56 (33-67) |
| Patient gender (M/F) | 59/41 |
| Serum M-protein |  |
| IgG, kappa | 25 |
| IgG, lambda | 18 |
| IgA, kappa | 16 |
| IgA, lambda | 5 |
| Light chain, kappa | 17 |
| Light chain, lambda | 13 |
| Other | 6 |
| Durie-Salmon stage |  |
| II | 14 |
| IIIA/B | 66/20 |
| ISS stage |  |
| I / II / III / NA | 29/44/23/4 |
| Cytogenetics^a^ |  |
| Standard risk/ High risk/NA | 40/22/38 |
| Myeloma bone disease on plain radiographs, yes/no | 62/48 |
| Creatinine at diagnosis, mg/dL, median (range) | 1.0 (1.0-12.0) |
| β2-microglobulin at diagnosis, mg/mL, median (range) | 3.5 (1.3-25.7) |
| Duration from diagnosis to ASCT, months, median (range) | 7.0 (2.9-12.3) |
| Pre-transplant induction therapy |  |
| Bortezomib-based | 15 |
| Thalidomide-based | 37 |
| Bortezomib/thalidomide-based | 47 |
| Others | 1 |
| Response to induction therapy (%) |  |
| CR | 43 |
| VGPR | 35 |
| PR | 22 |

CR, complete remission; F, female; ISS, International Staging System; NA, not available; M, male; PR, partial response; VGPR, very good partial response; SD, stable disease

^a^ High-risk cytogenetics was defined as hypodiploidy or deletion of chr13 on conventional cytogenetics or presence of t(4;14), t(14;16), -17p on fluorescent *in situ* hybridization and/or conventional cytogenetics. All other cytogenetic abnormalities were considered standard risk.
