# Supplementary figures and images for: Different role of circulating myeloid-derived suppressor cells in patients with multiple myeloma undergoing autologous stem cell transplantation
Source: J Immunother Cancer. 2019 Feb 7;7:35. doi: 10.1186/s40425-018-0491-y (PMC6367772; doi:10.1186/s40425-018-0491-y)

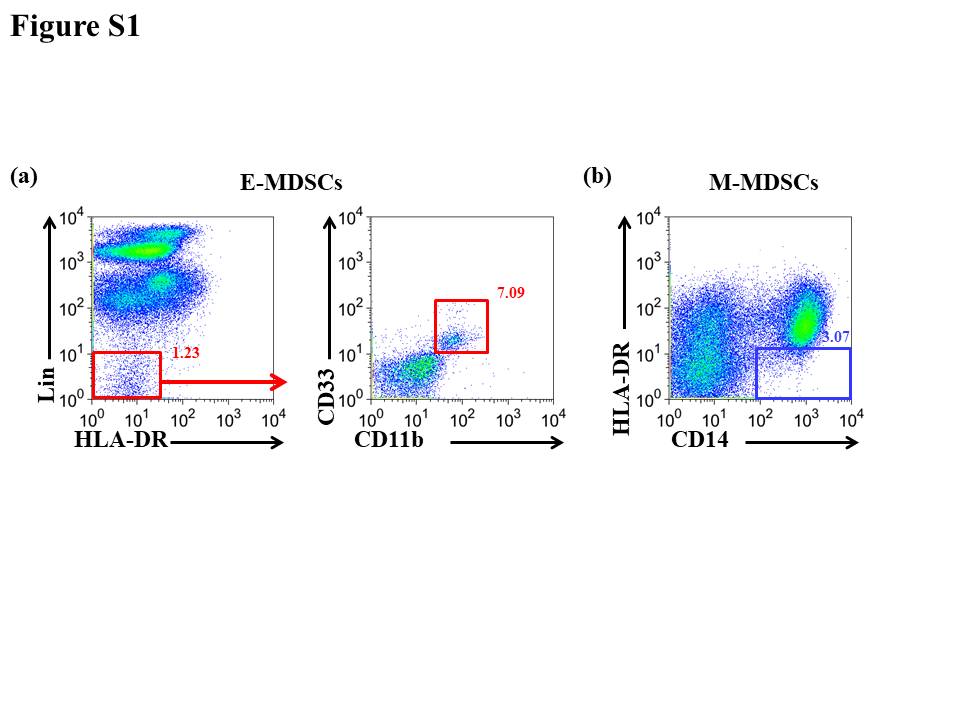

Supplement: Supplementary file 4 — Figure S1. Representative immunophenotypes of E- and M-MDSCs from PBMCs. Representative FACS plots of E- and M-MDSC phenotypes in PBMCs taken at the time of engraftment after ASCT. (TIF 180 kb) [file 40425_2018_491_MOESM4_ESM.tif]

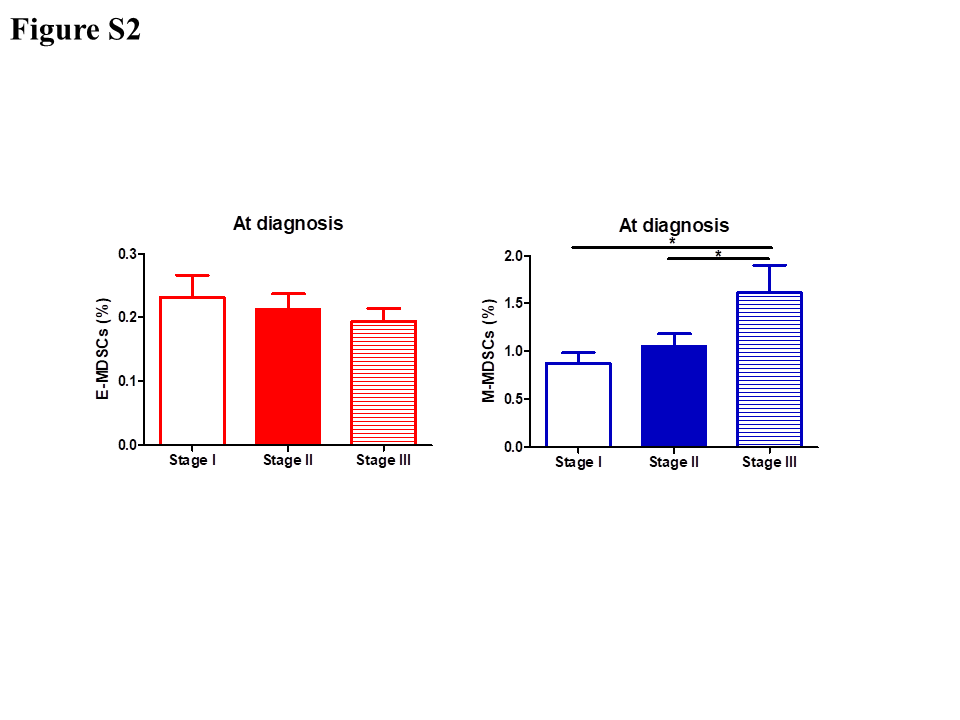

Supplement: Supplementary file 5 — Figure S2. Correlation between the frequency of E- and M-MDSC phenotypes at diagnosis and disease stage by the International Staging System. The frequency of E- and M-MDSC phenotypes at diagnosis was compared in the three groups (n = 56, 93, and 79 for stage I, II, and III, respectively). The data are presented as the mean ± SEM. *P < 0.05. (TIF 36 kb) [file 40425_2018_491_MOESM5_ESM.tif]

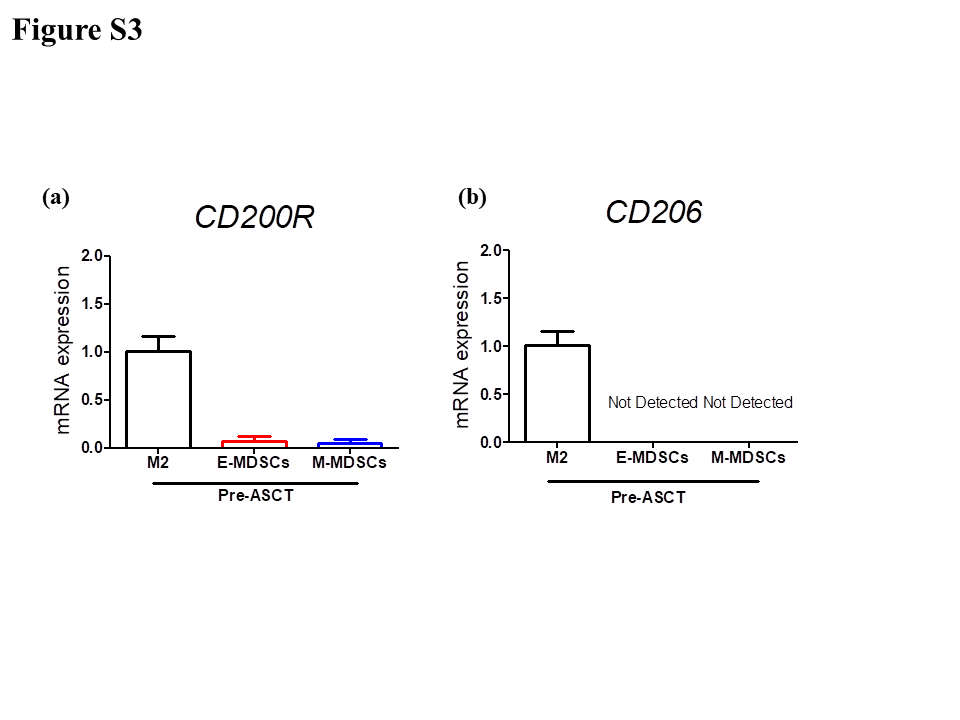

Supplement: Supplementary file 6 — Figure S3. Different macrophage gene expression between M2 polarized macrophages and pre-ASCT MDSCs. M2 macrophages, E- and M-MDSCs were isolated from PBMCs in pre-ASCT (n = 9). Expression of CD200R and CD206 was assessed in M2 macrophages and pre-transplant isolated MDSCs by qRT-PCR. (TIF 34 kb) [file 40425_2018_491_MOESM6_ESM.tif]

**Figure S4a**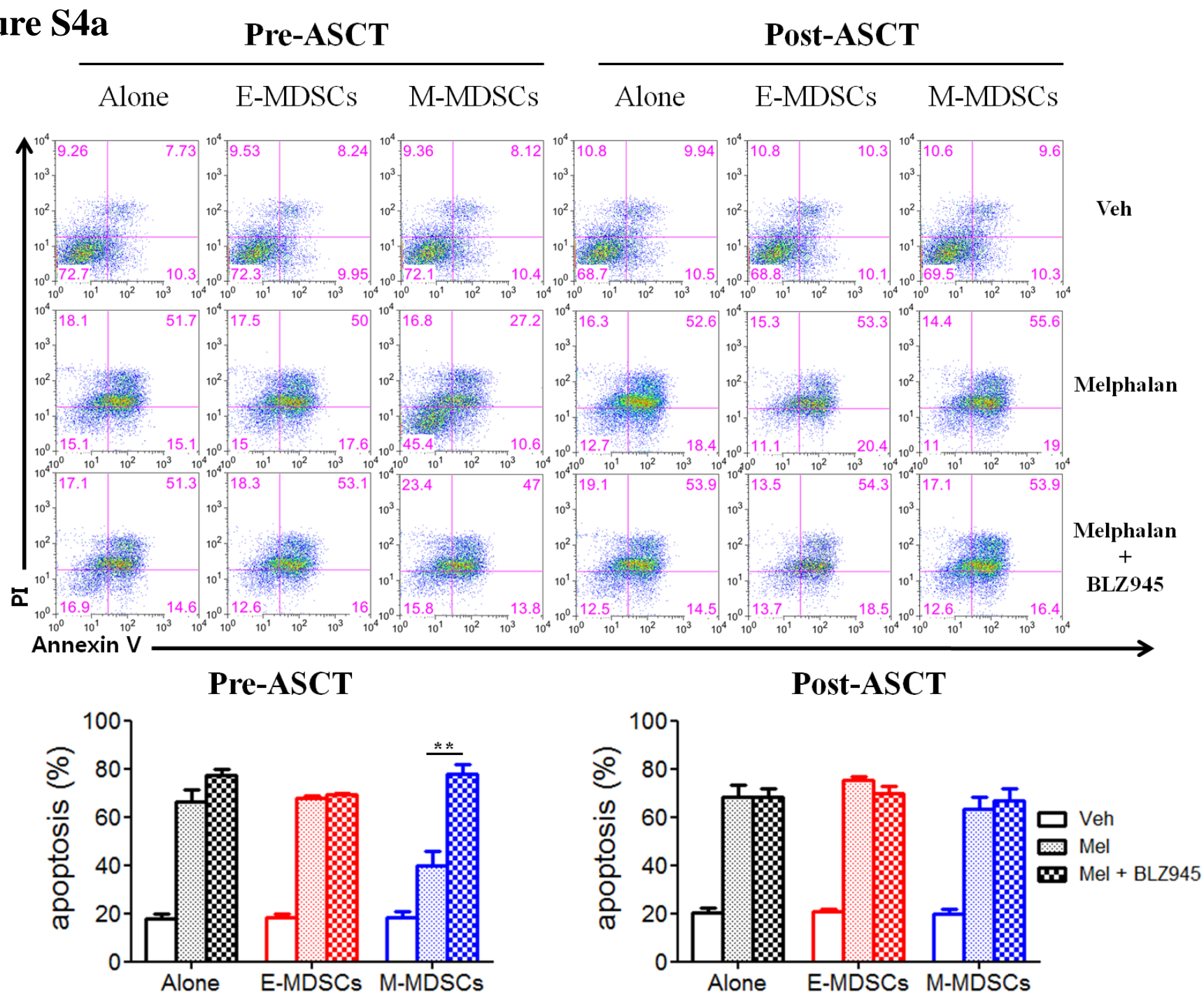

**Figure S4b**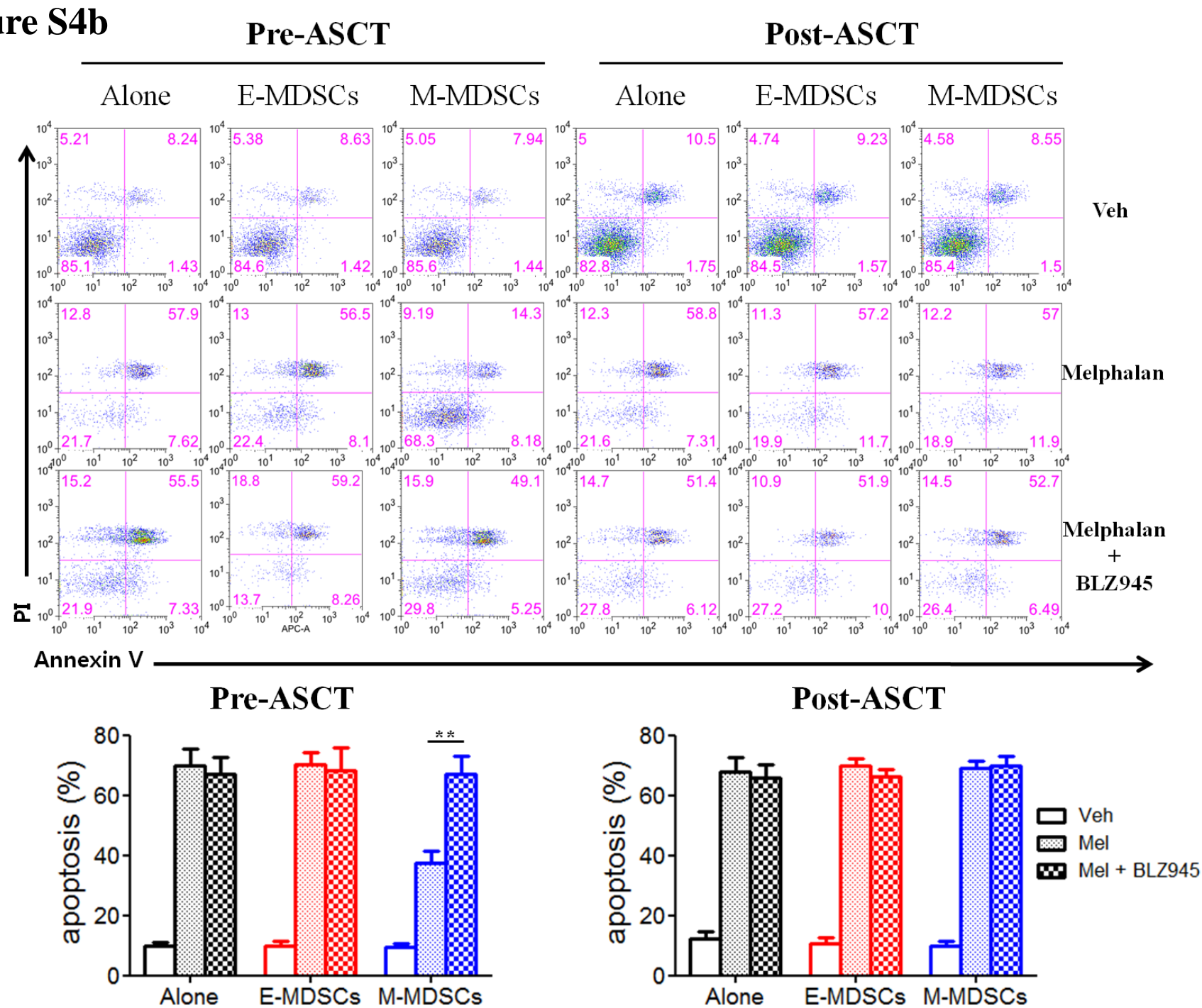

Supplement: Supplementary file 7 — Figure S4. The influence of CSF1R inhibition on melphalan-induced cytotoxicity attenuated by pre-ASCT M-MDSCs was assessed in the RPMI 8266 (a) and OPM2 cell lines (b). The same procedure as in Fig. 5 was carried out. (PDF 1259 kb) [file 40425_2018_491_MOESM7_ESM.pdf]
